# Comparison of Pharyngeal Airway between Mandibular Setback Surgery Patients (Skeletal Class III) and Nonsurgery Patients (Skeletal Classes I and II)

**DOI:** 10.1155/2019/5012037

**Published:** 2019-03-17

**Authors:** Jung-Hsuan Cheng, Chun-Ming Chen, Ping-Ho Chen, Szu-Ting Chou, Chin-Yun Pan, Yu-Chuan Tseng

**Affiliations:** ^1^Graduate Institute of Dental Sciences, School of Dental Medicine, Kaohsiung Medical University, Kaohsiung, Taiwan; ^2^Department of Orthodontics, Kaohsiung Medical University Hospital, Kaohsiung, Taiwan; ^3^Department of Oral and Maxillofacial Surgery, Kaohsiung Medical University Hospital, Kaohsiung Medical University, Taiwan; ^4^School of Dentistry, Kaohsiung Medical University, Kaohsiung, Taiwan

## Abstract

**Purpose:**

We investigated the pharyngeal airway dimensions and their correlations in patients who underwent mandibular setback surgery versus those who did not.

**Materials and Methods:**

One hundred and sixty cephalometric radiographs (120 patients) were obtained from patients with three skeletal malocclusion classifications: Class I and Class II in the nonsurgery group and Class III in the surgery group (preoperative and postoperative cephalograms). The following dimensions were measured: nasopharyngeal airway (NOP), uvulopharyngeal airway (UOP), shortest distance from the posterior tongue to the pharyngeal wall (TOP), and distance from the epiglottis to the pharyngeal wall (EOP). Paired* t* test, one-way analysis of variance, and Pearson correlation coefficients were used for statistical analysis.

**Results:**

Preoperatively, UOP and TOP of skeletal Class III patients (15.2 mm and 16.6 mm) were significantly larger than those of skeletal Class II (11.5 mm and 12 mm) and Class II (12.3 mm and 12.9 mm) patients, respectively. No differences were observed in EOP between the three skeletal patterns. The hyoid bone of Class III patients was significantly anterior to that of Class I/II patients. Furthermore, UOP had a moderate negative correlation with soft palate length. Postoperatively, no significant difference (UOP, TOP, EOP, soft palate width, and hyoid bone) was found between the skeletal classes.

**Conclusion:**

Preoperatively, UOP and TOP of skeletal Class III patients were significantly wider than those of skeletal Class I/II patients. Pre- and postoperatively, EOP did not exhibit significant differences among the three skeletal classifications. No differences were found in all postoperative pharyngeal airway dimensions between Class III patients and nonsurgery patients (Class I and Class II).

## 1. Introduction

The pharyngeal airway space has recently become an important issue in orthodontic treatment. Muto et al. [1] reported that the pharyngeal airway space in individuals with skeletal Class III malocclusion is larger than that in those with skeletal Class I or II malocclusion. Opdebeeck et al. [2] explored vertical facial patterns and found that patients with a long face had a narrow airway space compared with those with short faces. Orthodontic treatment only focuses on altering the cosmetic appearance of the smile rather than maintaining an appropriate airway space. After teeth retraction, the tongue is set back, which constricts the pharyngeal airway, leading to breathing problems [3].

However, the dimensions of the pharyngeal airway are affected not only by the growth of the maxilla and mandible but also by the positions of the tongue and hyoid bone. In 1992, Adamidis et al. [4] reported that the position of the hyoid bone in patients with skeletal Class III malocclusion was more anterior than that in patients with skeletal Class I malocclusion. Furthermore, the prevalence of Class III malocclusion is higher in Asians than in Caucasians [5]. Orthodontic treatment combined with orthognathic surgery is commonly performed in patients with skeletal Class III malocclusion. After the mandibular setback, the hyoid and tongue are accommodated backward, which causes the narrowing of the pharyngeal airway space [6]. For maintaining pharyngeal airway patency, it is essential to focus on changes in the postoperative positions of the mandible, tongue, and hyoid bone. The present study analyzed the airway space in three skeletal patterns and in patients with skeletal Class III who underwent mandibular setback surgery.

## 2. Materials and Methods

Cephalograms of the study participants (who were recruited from Department of Dentistry, Kaohsiung Medical University Hospital, Taiwan) were classified according to the skeletal relationship between the maxilla and the mandible, in which an ANB ≤ 0° was skeletal Class III, 0°-4° was skeletal Class I, and ≥ 4° was skeletal Class II. All lateral cephalograms were recorded in the natural head position and during the end-expiration stage, and patients were instructed not to swallow while the cephalograms were being* taken*. Patients with craniofacial symptoms or deformity or those who underwent other craniofacial surgeries or had sustained craniofacial injuries were excluded. The participants were divided into a nonsurgery group (skeletal Class I and Class II) and a surgery group (skeletal Class III).

One hundred and sixty cephalograms were obtained from 120 patients: the nonsurgery group comprised 40 patients (20 men and 20 women) and the surgery group comprised 40 patients (20 men and 20 women) who underwent both preoperative and 1-year postoperative cephalography. The mean age of the patients was 23.3 years in the skeletal Class I group, 25.4 years in the Class II group, and 20.7 years in the Class III group. All patients in the surgery group underwent preoperative orthodontic treatment and isolated intraoral vertical ramus osteotomy.

**Figure 1:**
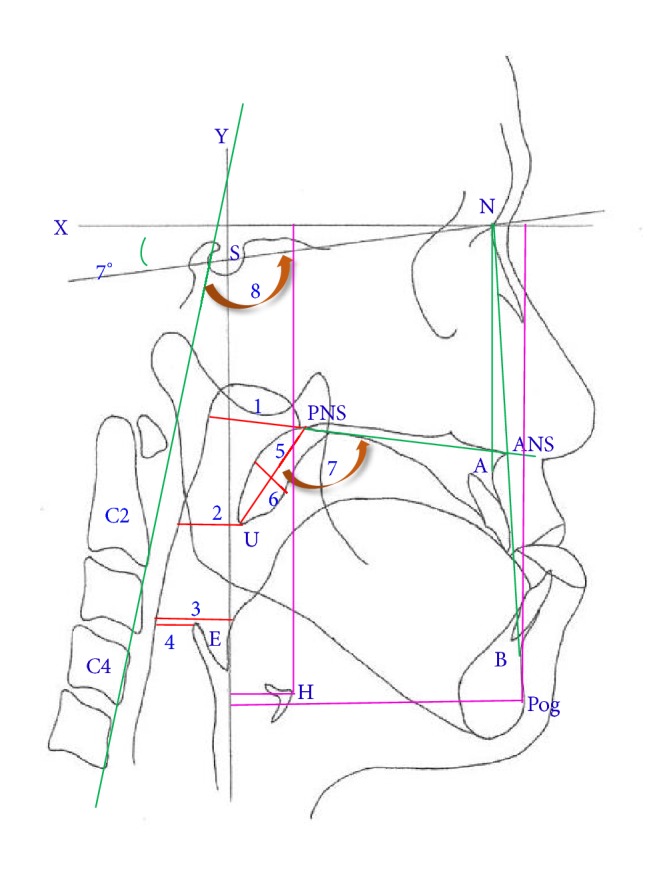
Cephalometric landmarks and linear measurements. Landmarks: nasion (N); sella (S); anterior nasal spine (ANS); point A; posterior nasal spine (PNS); point B; tip of uvula (U); inferoanterior point on the fourth cervical (C4); inferoanterior point on the second cervical (C2); most superior and anterior point on the hyoid bone (H); most superior point on the epiglottis (E); and Pogonion (Pog). The X-axis was constructed by drawing a line through the N, 7° above the SN line; the Y-axis was constructed by drawing a line through S, perpendicular to the X-axis. Linear distances: 1: NOP; 2: UOP, 3: TOP, 4: EOP, 5: SPL, 6: SPW. Angle measurement: 7: palatal angle, 8: C2C4-SN angle, ANB angle.

The following landmarks were identified on each cephalogram (Figure 1): nasion (N), sella (S), anterior nasal spine (ANS), point A, posterior nasal spine (PNS), point B, tip of uvula (U), inferoanterior point on the fourth cervical vertebra (C4), inferoanterior point on the second cervical vertebra (C2), most anterosuperior point on the hyoid bone (H), the most superior point on the epiglottis (E), and Pogonion (Pog). Linear and angular measurements of the following were taken: nasopharyngeal airway (NOP [ANS-PNS plane intersecting the pharyngeal wall]); distance from the tip of uvula to the pharyngeal wall (UOP, perpendicular to the y-axis); shortest distance from the posterior tongue to the pharyngeal wall (TOP); distance (parallel to the x-axis) from the epiglottis to the pharyngeal wall (EOP); width of the soft palate (SPW); length of the soft palate (SPL); ANB angle; palatal angle; and C2C4-SN angle: angle between the C4C2 line and SN line. We applied G*∗*Power version 3.1.9.2 to estimate the sample size (Franz, Universitat Kiel, Germany) (1). With power (1-*β*) of 90%, *α* of 0.05, and the estimated effect size of 0.73, the total sample size calculated was only 30—which was 10 for each group. In this study, we recruited 120 patients to achieve sufficient power of at least 90%.

Data were processed using SPSS version 20 (IBM Corporation, Armonk, NY, USA). Multiple comparisons between groups were performed using one-way analysis of variance. Tukey's honestly significant difference test was used for post hoc testing. Pearson correlation coefficients were used to analyze associations between pharyngeal airway depth, mandibular position, head position, and changes in the hyoid bone position. The paired* t* test was used to compare preoperative and postoperative values of skeletal Class III patients. Differences were considered to be statistically significant at* p* < 0.05. The present study was approved by the Institutional Review Board of Kaohsiung Medical University Hospital (KMUHIRB-E(II)-20180200).

## 3. Results

In Table 1 (preoperative data), the C4C2-SN angle and palatal angle of Class III patients (96.3° and 118.3°, respectively) were significantly smaller than those of Class II (107.1° and 125.9°, respectively) and Class I (106.4° and 123°, respectively) patients. SPL was significantly shorter in Class III patients (32.7 mm) than in Class II (37.3 mm) and Class I (36 mm) patients. UOP (15.2 mm) and TOP (16.6 mm) of Class III patients were significantly wider than those of Class II (11.5 mm and 12 mm, respectively) and Class I (12.3 mm and 12.9 mm, respectively) patients. EOP revealed no significant differences between the three skeletal classes. The Pog was significantly more anteriorly placed in Class III (79.6 mm) than in Class II (63.6 mm) and Class I (69.5 mm) patients; however, no significant differences were observed in the vertical Pog position between the three skeletal types. The hyoid bone was significantly more anteriorly placed in Class III (23.1 mm) than in Class II (14.1 mm) and Class I (17.3 mm) patients; however, no significant differences were observed in the vertical hyoid bone position between the three skeletal types.

Pearson correlation analysis of the four airway lengths and various skeletal characteristics is shown in Table 2. Skeletal patterns showed a significant correlation with UOP, TOP, and EOP. The palatal angle presented a significant correlation with all pharyngeal airway dimensions. UOP had a significant moderate negative correlation (*r* = -0.405) with SPL. The C4C2-SN angle and hyoid bone (horizontal and vertical positions) showed no significant correlation with any pharyngeal airway dimensions. Surgical changes in patients with Class III are summarized in Table 3. Pog was 10.7 mm backward significantly. The hyoid bone was 5.7 mm backward and 2.1 mm downward significantly. Patients had a significantly increased C4C2-SN angle, palatal angle, and soft palate length. Pharyngeal airway dimensions (UOP, TOP, and EOP) showed a significant reduction (2.7, 3.4, and 1.6 mm, respectively).

## 4. Discussion

Elham and Susan [7] reported that the uvuloglossopharyngeal airway space in patients with skeletal Class II malocclusion is affected by the horizontal positions of the maxilla and mandible. In our study, skeletal patterns had a significant correlation with UOP, TOP, and EOP. However, the growth of the nasopharynx (NOP) was similar among the three skeletal patterns. Relevant pharyngeal airway muscles are connected to the soft palate and the tongue [8, 9]. Moreover, the hyoid bone and its associated muscle tissues play crucial roles in maintaining pharyngeal airway spaces [10, 11]. Different mandible positions also often lead to different hyoid bone positions. Therefore, the size of the oropharyngeal space may be affected by the positions of the soft palate, tongue, hyoid bone, and mandible.

In this study, palatal angles had a significant positive correlation (*r* = 0.390) with NOP and a significant negative correlation to UOP (*r* = -0.243), TOP (*r* = -0.274), and EOP (*r* = -0.196). SPL had a significant positive correlation (*r* = 0.190) with NOP and a significant negative correlation with UOP (*r* = -0.405) and TOP (*r* = -0.180). SPW had a significant positive correlation (*r* = 0.183) with UOP. This finding means that UOP was primarily affected by the soft palate, especially SPL. SPL and the palatal angle were significantly longer and wider, respectively, in patients with Class II malocclusion than in patients with Class III malocclusion. This suggests that mandibular protrusion in Class III leads to a more anterior palatoglossus muscle and a smaller palatal angle. This result may be because patients with severe skeletal Class III malocclusion likely have their uvula slightly pulled toward the anterior by the palatoglossus muscle, thereby indirectly creating a larger UOP. Therefore, UOP presented significant differences in this study: Class III > Class II or Class I.

A study examining the correlation between the lengths of separate airway dimensions and the anatomical structure [12] reported that the oropharyngeal airway dimensions were correlated with the position of the tongue and hyoid bone. TOP was greater in patients with skeletal Class III malocclusion than in those with Class II and Class I because patients with skeletal Class III had more anterior positions of the tongue and the hyoid bone, thereby increasing the distance between the dorsum of the tongue and the posterior pharyngeal wall. Even the hyoid bone's position in Class III patients was significantly anterior to that in Class I and Class II patients. However, our study yielded a different finding: the position of the hyoid bone (horizontal and vertical) was not significantly correlated with the pharyngeal airway length. This means that the growth of the mandible primarily led to increased dimensions of the oropharyngeal airway (UOP and TOP). No significant differences were found in EOP, suggesting that it did not vary among the skeletal types and that it was unaffected by the skeletal relationship between the maxilla and mandible.

Solow et al. [13] found that the greater the craniocervical angle, the more retruded the mandible. By contrast, the more protruded the mandible, the more protruded the hyoid bone, leading to a narrower craniocervical angle. Because the airway was widened, no head raising was required to ensure that the airway remained sufficiently wide for breathing. Moreover, we found that the C4C2-SN angle had no significant correlation with any pharyngeal airway dimension and that the C4C2-SN angle of Class III (96.3°) was significantly smaller than that of Class II (107.1°) and Class I (106.4°).

*Muto et al. [14]* studied the effect of mandibular setback surgery on the pharyngeal airway and found that the average reduction in airway length at the soft palate and posterior tongue area was 2.6 mm and 4.0 mm, respectively, and the palatal angle increased by 4°.* Achilleos et al. [15]* reported that the craniocervical angle increased by 3.2° after surgery. Our findings are consistent with the results of these studies: pharyngeal airway dimensions (UOP, TOP, and EOP) showed a significant reduction (2.7, 3.4, and 1.6 mm, respectively) after surgery, and the C4C2-SN and palatal angles increased by 3.6° and 4.4°, respectively, after surgery. To enable smooth breathing, the head rises naturally to increase the airway space. Our results revealed minimum changes in the NOP, indicating virtually no effect due to mandibular setback surgery. Backward movement of the mandible may cause airway narrowing, which depends on not only the amount of mandibular setback but also the adjustment of the surrounding structures such as the hyoid bone. In the present study, a significant setback (10.7 mm) was observed with Pog; and to compensate for this, the hyoid bone moved 5.4 mm backward and 2.1 mm downward. Thus, the compression of the pharyngeal airway was relieved, and respiratory patency was maintained.

The causes of breathing difficulty or apnea primarily occur at the UOP and TOP dimensions. Notably, rotation (clockwise or counterclockwise) of the mandibular setback brings about a change in the pharyngeal airway [16]. In clockwise rotation, the mandibular symphysis descends, but the mandibular body and ramus rise, which causes the tongue base to compress the TOP airway, thereby reducing much of the TOP airway space. Nevertheless, the uplifting function of the tongue base counterbalances the setback and downward position of the hyoid bone. This situation is reversed in a counterclockwise rotation. Therefore, further study is required to investigate the effects on the pharyngeal airway after clockwise and counterclockwise rotation following mandibular setback procedures.

In conclusion, UOP and TOP of patients with skeletal Class III malocclusion were significantly wider than those of patients with skeletal Class II and Class I malocclusion. EOP did not exhibit significant differences among the three skeletal classifications. UOP and TOP had a significant positive correlation with skeletal classifications and a significant negative correlation with the palatal angle and SPL. However, the position of the hyoid bone showed no significant correlation with the pharyngeal airway. The pharyngeal airway lengths of postoperative patients (Class III) exhibited no differences compared with nonsurgery patients (Class II and Class I).

## Figures and Tables

**Table 1:** Patients characteristics in the skeletal classifications.

Variables	Class I (n= 40)		Class II (n= 40)		Class III (n= 40)		Preoperation intergroup comparison		
	Mean	SD	Mean	SD	Mean	SD	F	*P* value	Significant
C4C2-SN angle	106.4	6.44	107.1	6.59	96.3	7.31	31.158	< 0.001	II > III, I > III
Palatal angle	123.0	5.33	125.9	5.50	118.3	6.71	17.005	< 0.001	II > III, I > III
Soft palate length	36.0	4.32	37.3	4.90	32.7	3.04	12.794	< 0.001	II > III, I > III
Soft palate width	8.6	1.62	8.4	1.62	9.6	1.63	5.762	0.004	III > II, III > I
Pharyngeal airway									
NOP	23.5	3.72	25.3	2.79	23.2	3.00	4.909	0.009	II > III, II > I
UOP	12.3	3.31	11.5	2.49	15.2	3.08	17.37	< 0.001	III > II, III > I
TOP	12.9	3.61	12.0	3.31	16.6	4.50	16.253	< 0.001	III > II, III > I
EOP	7.9	2.41	7.9	2.59	9.6	4.39	3.674	0.052	–
Pogonion									
Horizontal	69.5	7.92	63.6	6.69	79.6	9.34	40.093	< 0.001	III > I > II
Vertical	124.6	9.14	124.0	6.62	121.6	9.25	1.392	0.253	–
Hyoid									
Horizontal	17.3	7.86	14.1	7.13	23.1	9.57	12.194	< 0.001	III > II, III > I
Vertical	123.1	10.60	122.8	10.65	123.7	11.20	0.068	0.934	–

n: number of patient

I: Class I, II: Class II, III: Class III

NOP: nasopharyngeal airway, UOP: uvulopharyngeal airway

TOP: shortest distance from posterior tongue to pharyngeal wall, EOP: distance from epiglottis to pharyngeal wall

Statistically significant,* p <* 0.05

–: Not significant

**Table 2:** Preoperation pharyngeal airways in the skeletal classifications (Pearson test).

Total	Classifications	C4C2-SN angle	Palatal angle	SPL	SPW	Hyoid H	Hyoid V
NOP	-0.038	0.033	0.390*∗*	0.190*∗*	-0.047	-0.173	-0.076
UOP	0.354*∗*	-0.136	-0.243*∗*	-0.405*∗*	0.183*∗*	-0.031	0.025
TOP	0.350*∗*	-0.085	-0.274*∗*	-0.180*∗*	0.129	0.133	0.132
EOP	0.211*∗*	-0.082	-0.196*∗*	-0.097	0.021	0.132	0.132

SPL: Soft palate length; SPW: Soft palate width; Hyoid H (Horizontal); Hyoid V (Vertical)

**∗**: Statistically significant, *p* < 0.05; very weak (0 – 0.19), weak (0.20 – 0.39), moderate (0.40 – 0.59), strong (0.60 – 0.79), and very strong (0.80 – 1.0).

**Table 3:** Patients characteristics (Class III) in the surgical changes.

Variables	Surgical changes			Postoperation intergroup comparison		
	Mean	SD		F	*P* value	Significant
C4C2-SN angle	3.6	5.17	*∗*	12.362	< 0.001	II > III, I > III
Palatal angle	4.4	6.21	*∗*	4.325	0.015	II > III
Soft palate length	1.3	3.33	*∗*	5.258	0.007	II > III
Soft palate width	-0.4	1.68	–	2.584	0.080	–
Pharyngeal airway						
NOP	0.4	2.21	–	3.626	0.030	II > I
UOP	-2.7	2.18	*∗*	1.309	0.274	–
TOP	-3.4	3.77	*∗*	1.116	0.331	–
EOP	-1.6	3.15	*∗*	0.020	0.980	–
Pogonion						
Horizontal	-10.7	4.65	*∗*	6.289	0.003	III > II, I > II
Vertical	-0.7	3.22	–	2.202	0.115	–
Hyoid						
Horizontal	-5.4	5.14	*∗*	2.271	0.108	–
Vertical	2.1	6.30	*∗*	0.977	0.379	–

I: Class I, II: Class II, III: Class III

NOP: nasopharyngeal airway, UOP: uvulopharyngeal airway

TOP: shortest distance from posterior tongue to pharyngeal wall

EOP: distance from epiglottis to pharyngeal wall

*∗*: Statistically significant, *p* < 0.05; –: Not significant

## Data Availability

The data used to support the findings of this study are included within the article. The data used to support the findings of this study are available from the corresponding author upon request.
